# Clinical Trial Highlights – Interventions Promoting Physical Activity in Parkinson’s Disease

**DOI:** 10.3233/JPD-239001

**Published:** 2023-05-09

**Authors:** Thomas H. Oosterhof, Sabine Schootemeijer, Nienke M. de Vries

**Affiliations:** Department of Neurology, Center of Expertise for Parkinson & Movement Disorders, Radboud University Medical Center, Nijmegen, Netherlands

## Abstract

Despite increasing evidence on exercise in Parkinson’s disease (PD) it remains unclear what type and intensity of exercise are most effective. Currently, most evidence favors moderate- to high-intensity aerobic exercise for its positive effects on motor symptoms as well as disease modifying potential. On the other hand, observational studies have shown that the sheer volume of exercise matters as well, independent of intensity. So far, the efficacy of the volume of exercise has not been confirmed by randomized controlled trials (RCTs). Here, we provide an overview of the ongoing RCTs that promote physical activity in daily life in PD. We found seven RCTs with sample sizes between 30 and 452 and a follow-up between 4 weeks and 12 months. Steps per day is the most commonly reported primary outcome measure. The ongoing RCTs will provide evidence for feasibility, whereafter the PD research field is ready for a next step and to explore the effect of physical activity on disease progression and PD symptoms.

## INTRODUCTION

There is increasing evidence and marked interest for non-pharmacological interventions in people with Parkinson’s disease (PD), especially exercise [1]. Moderate- to high-intensity exercise has beneficial effects on motor symptoms [2, 3] with seemingly the most potent effect from high-intensity exercise [2, 4]. Exercise also positively impacts non-motor symptoms such as depression [5, 6] and cognition [7, 8]. Even though clinical trials mostly apply a high-intensity exercise intervention, this type of exercise could be challenging for people with a neurological disease like PD. They may be confronted with multiple barriers due to motor symptoms or non-motor symptoms, such as fatigue and apathy [9]. Different studies also indicate a potential disease-modifying effect from low-intensity exercise or an increase of sheer volume of physical activity. For example, a recent systematic review and meta-analysis shows that low-intensity exercise improves neuroplasticity in patients with neurological disease, including PD, with an equal effect compared to high-intensity exercise [10]. Moreover, observational studies indicate an inverse association between the volume of physical activity and the incidence of PD [11–16] and show that people with PD who are more active, have a slower deterioration of PD symptoms (e.g. gait stability, activities of daily living, and processing speed) [17]. Even reduced mortality rates have been reported, accompanied by a dose-response association, regardless of the intensity [18]. Nonetheless, clinical trials focusing solely on the effect of a higher volume of low-intensity exercise are lacking.

Based on the converging observational evidence, increasing the volume of physical activity in daily life is an interesting approach to explore further. Increasing the volume of physical activities in daily life, such as walking, would be a very accessible option for those who are not able to engage in high-intensity exercise due to impairments or who don’t have access to sport facilities. Here, we aim to review to what extent the existing evidence from observational studies is now being tested further in randomized controlled trials (RCTs). For this purpose, we provide an overview of the ongoing RCTs that promote the volume of physical activities in daily life in PD.

## OVERVIEW OF RANDOMIZED CONTROLLED TRIALS OF INTERVENTION PROMOTING PHYSICAL ACTIVITY IN DAILY LIFE

We searched Clinicaltrials.gov for interventional studies in PD with the terms ‘’physical activity” and “Parkinson’s disease”. A study was only included if its status was ‘’not yet recruiting”, ‘’recruiting” or ‘’active, not recruiting”. A study was excluded if it was not a RCT, if a combined intervention was studied, if the intervention consisted of another type of intervention (i.e. medication, a structured exercise intervention or a dietary intervention).

**Fig. 1:**
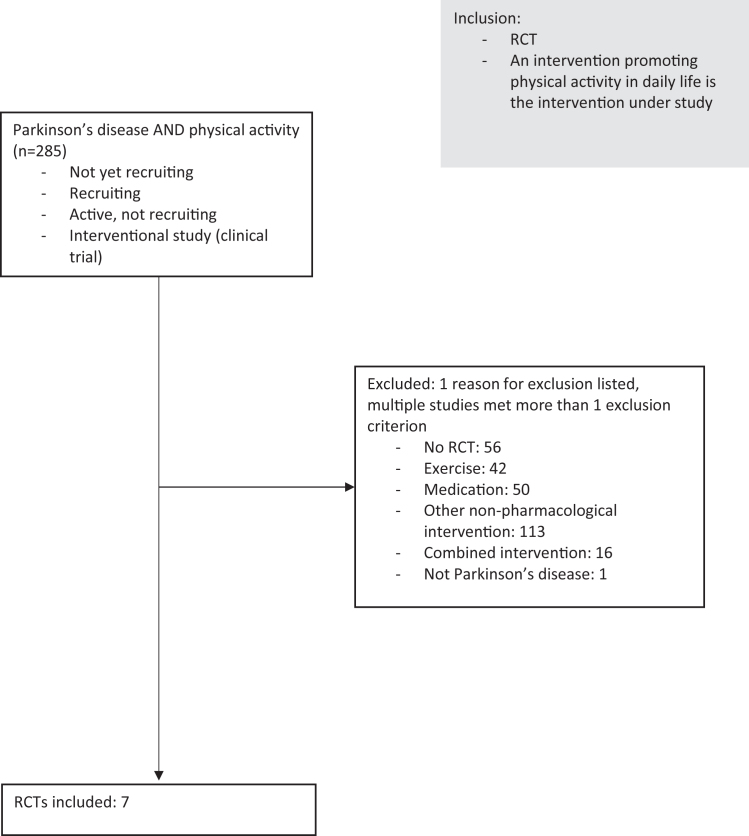
Flowchart. RCT = randomized controlled trial.

If the people under study did not have Parkinson’s disease, the study was also excluded (Fig. 1). This resulted in the inclusion of 7 studies: Pre-Active PD, STEPS-PD, Amped-PD, MoTIvatE, STEPWISE, KEEP and WHIP-PD. The key elements of the included trials are summarized in Table 1. In the following paragraphs, we will discuss some general characteristics of the studies.

**Table 1:** Overview of clinical trials on interventions promoting physical activity

	Study	Sponsor	ID	Promotional intervention	Phase	Estimated enrolment	Estimated completion date	IPD sharing statement
1.	Pre-Active PD	San Jose State University	NCT05308238	Telehealth-delivered physical activity behavior change intervention	N/A	50	March 2024	Not reported
2.	STEPS-PD	Stiftelsen Stockholms Sjukhem	NCT05510739	eHealth based motor cognitive home training	N/A	100	March 2024	No
3.	AMPED-PD	Boston University Charles River Campus	NCT05421624	Adaptive rhythmic auditory stimulation (RAS)	N/A	44	April 2024	No
4.	MoTIvatE	University of Pennsylvania	NCT04051151	Gamification delivered exercise intervention	N/A	84	December 2023	Undecided
5.	STEPWISE	Radboud University Medical Center	NCT04848077	mHealth smartphone based exercise solution	N/A	452	July 2026	Yes
6.	KEEP	University of Cambridge	NCT05253040	Co-designed online education modules and group discussion about exercise	N/A	30	June 2023	Undecided
7	WHIP-PD	Boston University Charles River Campus	NCT03517371	mHealth delivered exercise and cognitive-behavioral program	2	148	May 2024	No

Estimated enrolment = number of participants, IPD = Individual Participant Data. N/A = not applicable.

### Population

Samples sizes vary between 30 and 452. Four studies include people with PD who are older than 18 years of age, AMPED-PD includes people older than 40 years, STEPS-PD older than 50 years and MoTIvatE includes all ages, including children. Five studies recruit participants with Hoehn and Yahr equal to or less than 3, while disease severity is not specified in two studies. KEEP includes only people with PD with an early diagnosis within 12 months prior to inclusion.

### Interventions

All studies deliver an intervention to promote home-based physical activity in daily life, with walking being the most prevalent type of activity. Other studies included activities of the participants’ preference. The degree of supervision varies from active supervised online discussion to an unsupervised, digital intervention. All interventions are home-based, except for WHIP-PD, which is the only study adding in-person in-clinic physiotherapy sessions. Most studies apply different cognitive-behavioral elements such as feedback on performance, self-monitoring and goal setting. KEEP solely focuses on education and AMPED-PD highlights the effect of music on physical activity. The partner is included as motivator in MoTIvatE.

The encouraged frequency of physical activity varies from no specific target frequency to 5 to 7 days per week. Only limited information about the exact details of the interventions is reported in the study registrations. The WHIP-PD study describes the intervention in most detail and for this study, a design article is also available [19].

### Primary and secondary outcome measures

Physical activity quantified as steps per day is the most prevalent primary outcome (four studies), and is included as a secondary outcome in the other three studies. Three studies include a primary outcome attempting to assess a measure of the intensity in which these steps are taken. Other primary outcomes include feasibility, compliance, recruitment rate, gait quality and subjective physical activity levels. Five studies report more than one primary outcome. In addition, a variety of secondary outcomes such as gait parameters, quality of life, dual-tasks, balance, system usability, knowledge of exercise, motor symptoms and non-motor symptoms such as depression and cognition are assessed. Information about power and a sample size calculation is only reported by STEPWISE and WHIP-PD.

### Follow-up duration

The duration of the interventions varies from 4 weeks to 12 months. Three studies also include follow-up assessments post-intervention varying from 2 weeks to 6 months after termination of the intervention.

### Blinding

Four studies are single-blinded and two studies are double-blinded. MoTIvatE is the only unblinded, open-label study.

### Compliance

KEEP is the only study reporting compliance as an outcome measure, defined as the percentage of education modules completed.

### IPD sharing statement

Individual participant data (IPD) will be shared by one out of seven studies only.

## DISCUSSION

We provided an overview of the ongoing RCTs studying promotion of the volume of physical activity in persons with PD. Overall, all studies focus on proving the feasibility (i.e., study whether it is possible to structurally increase the volume of physical activity in people with PD) rather than studying the effectiveness on symptoms, disease progression or disease modifying markers. The nature and design of the RCTs varied. The results of these studies will provide us with essential knowledge on how to shape future studies on the efficacy of increasing the sheer volume of physical activity in people with PD.

A lifestyle change is hard to maintain [20], and these studies will inform us what type of intervention could help people with PD to engage (and keep engaging) in a physically active lifestyle. Digital and remotely delivered interventions will be markedly interesting for its presumably easy accessibility. The WHIP-PD and STEPWISE trials will be interesting in particular, with adequate power and long follow-up. These two studies will provide insights into the feasibility of mHealth delivered support and motivation. Studies on the maintenance of an active lifestyle over a longer period of time are necessary to study effectiveness on PD progression or a potential disease-modifying effect. Three studies in this overview will study the efficacy on Parkinson related symptoms in exploratory analyses. However, these will be evaluated as secondary outcome and not as primary outcome, which may hamper solid conclusions since they are not powered to measure an effect on these outcomes. In the present studies, the effect on putative underlying mechanisms of disease progression such as imaging or biochemical changes are not taken into account. The effect of the sheer volume of physical activity on these biomarkers is unknown, but may be interesting to consider, since higher intensity exercise has shown to induce adaptive cerebral plasticity, as demonstrated using functional and structural magnetic resonance imaging [21]. In addition, moderate-intensity exercise in persons with PD may also alter a variety of blood biomarkers related to inflammation or neuroplasticity [22].

The studies presented here are characterized by several strengths and limitations. Overall, a strength is that all studies include a remote intervention, which is highly relevant because it is easily accessible for large groups of people. In addition, the chosen outcomes assessments are done with validated in-clinic tests or remotely assessed with accelerometers, which are commonly used to evaluate step counts, as a derivative for the volume of physical activity. Outcomes will also be generalizable for an early stage of Parkinson’s disease (i.e. H&Y 3 or less). This is the group of interest who might benefit most from the volume of physical activity and in whom the risk of falling due to increasing activity will be less than those in a further stage. Limitations of these studies are the small sample sizes and the fact that information about power calculations is mostly lacking. In addition, more than half of the included studies have short follow-up periods equal to or less than 10 weeks, which will make it harder to draw conclusions regarding feasibility over a longer period of time. Moreover, only two studies are at least double-blinded, although we are aware that implementing proper blinding in non-pharmacological studies is challenging. Individual participant data (IPD) will be shared by only one out of sevens studies. Despite the heterogeneity of the interventions, data sharing can benefit future studies investigating or incorporating similar ways to promote the volume of physical activity in daily life.

Observational data show an inverse association between the volume of physical activity and the progression of PD symptoms, but clinical trials are still lacking. The insights that will result from the trials currently being performed will not be able to answer effectiveness questions, but will certainly help in designing such clinical trials in the future. Observational data also show that exercise may postpone or even prevent PD [11–16]. An interesting approach besides focusing on people diagnosed with PD, would be to evaluate a putative disease modifying effect of physical activity on the course of disease in a (presumably) prodromal phase. The prevalence of PD is expected to grow exponentially in the coming years [23]. Potential beneficial effects of the volume of physical activity in a prodromal phase could prove to be useful to alter this alarming course.

In conclusion, a variety of interventions promoting the volume of physical activity are currently under study and we look forward to their results. While the benefits of moderate- to high intensity exercise have been established unequivocally, these RCTs mainly focus on feasibility. In the field of moderate to high intensity exercise, we are waiting for the results of the SPARX 3 study [24] (NCT04284436) to address the question of the impact of exercise intensity on the rate of PD progression in early disease. The field of PD deserves and also needs such future trials promoting the volume of physical activity with sufficient power and long follow-up. These studies should evaluate the effect on disease progression markers, such as metabolic changes and imaging, in people in different stages of PD or even consider including people in a prodromal phase. Besides the volume of physical activity, we need more evidence on the effectiveness of the broad spectrum of lifestyle interventions (i.e. nutrition, or stress management) for people with PD. Given the shared complexity and methodological challenges related to studying lifestyle interventions, studies on physical activity may serve as an inspiring example.

## DETAILS OF RANDOMIZED CONTROLLED TRIALS OF INTERVENTION PROMOTING PHYSICAL ACTIVITY IN DAILY LIFE

**San Jose State University - Pre-Active PD: Looking at Physical Activity Behavior Change in Parkinson’s Disease**

***Title***: Pre-Active PD: a Randomized Control Trial (RCT) Pilot Study to Improve Self-management of Physical Activity (PA) Routines in Adults With Early-stage Parkinson’s Disease (PD)

***Phase***: N/A

***Objective***: Determining the feasibility of a telehealth-delivered physical activity behavior change intervention and the effects on long-term physical activity levels

***Status***: Recruiting

***Clinicaltrials.gov ID***: NCT05308238

***Sponsor***: San Jose State University

***Collaborators***: Teachers College, Columbia University

***Estimated enrolment***: 50

***Estimated completion date***: March 2024

***Study design***: This pilot study is a single-blind (outcomes assessor) randomized controlled trial. Participants (*n* = 50) are randomly assigned (parallel assignment 1: 1) to an intervention or control group. The intervention consists of four months of telehealth coaching sessions and brief email or text check-ins supported by a Fitbit and an Engage PD workbook to increase their physical activity level. Assessments are performed at baseline, 4 months and 6 months (2 months post-intervention). The trial recruits people diagnosed with PD, aged 18–85 years, Hoehn and Yahr stage I or II, who are ambulatory for indoor and outdoor mobility without assistance or assistive device, successful completion of Physical Activity Readiness Questionnaire (PAR-Q), or medical clearance from the general practitioner. People with any musculoskeletal injury that would prevent exercise, other neurological disorders or people who already engage in aerobic exercise for more than 30 minutes or more than 5 days per week, are excluded.

***Intervention***: Six telehealth coaching sessions and 8 e-mail or text check-ins over the course of 4 months supported by a Fitbit and Engage PD workbook. The control group receives 6 hours of online educational videos regarding disease management and one, optional, telehealth coaching session supported by an Engage PD workbook.

***Outcome measures***: Primary: Feasibility (adherence and retention) [4 months], physical activity (Brunel Lifestyle Physical Activity Questionnaire and Moderate-Vigorous Physical Activity Using Actigraph) [baseline, 4 and 6 months]. Secondary: Timed-up-and-go (TUG) [baseline, 4 and 6 months], 30 second sit-to-stand [baseline, 4 and 6 months], Modified 2 minute step test [baseline, 4 and 6 months], Perceived functional ability questionnaire [baseline, 4 and 6 months], Modified Canadian occupational performance measure [baseline, 4 and 6 months], Patients’ global impression of change scale [baseline, 4 and 6 months], Life space assessment [baseline, 4 and 6 months], Post-intervention acceptability questionnaire [4 months], Intervention implementation [4 months], Modified physical activity rating (PA-R) questionnaire [baseline, 4 months], Perceived autonomy support healthcare climate questionnaire [4 months]

***Comments***: This trial investigates whether telehealth coaching can promote physical activity in daily life. Four primary outcome measures are reported, which is unusual, in addition to many secondary outcomes. The nature of the ‘Engage PD workbook’ is not reported, which makes it unclear what this entails. The inclusion criterion of successful completion of the PAR-Q is not specified. An interesting outcome will be the change in physical activity 2 months post-intervention, possibly indicating a reflection of the effect in the longer term and the possible initiation of behavioral change. Although, given the fact that this study is a pilot with a relatively small sample size and the relatively short intervention and follow-up period, this may hinder a conclusion on efficacy. This study will give insights whether it is feasible to use telehealth coaching to promote physical activity in daily life, which can inform larger and longer future studies.

***Results***: The estimated study completion date is in March 2024

***References***: -

**Stiftelsen Stockholms Sjukhem - Support for Physical Activity in Everyday Life With Parkinson’s Disease (STEPS-PD)**

***Title***: Support for Physical Activity in Everyday Life With Parkinson’s Disease Using eHealth Technology

***Phase***: N/A

***Objective***: Study the efficacy of an eHealth based motor-cognitive home training on physical activity levels

***Status***: Not yet recruiting

***Clinicaltrials.gov ID***: NCT05510739

***Sponsor***: Stiftelsen Stockholms Sjukhem

***Collaborators***: Karolinska Institutet

***Estimated enrolment***: 100

***Estimated completion date***: March 2024

***Study design***: This is a double-blind randomized controlled trial. Participants (*n* = 100) are randomly assigned (parallel assignment 1: 1) to a 10-week eHealth based motor-cognitive home training program with cognitive behavioral strategies or a paper-based home exercise program without support. Assessments are performed at baseline and after the intervention (10 weeks). The trial recruits people diagnosed with PD, Hoehn and Yahr II-III, age 50 years or older, who are able to walk indoors without mobility aid and who can continually walk for at least 6 minutes with or without a walking aid. People who have cognitive impairment (Montreal Cognitive Assessment≤21 points), major problems with freezing and/or more than two falls in the month previous to inclusion, other neurological, orthopedic or cardiovascular disease impeding performing unsupervised exercise, impaired vision or communication or have no internet connection at home are excluded.

***Intervention***: Ten week eHealth motor-cognitive, individualized and progressive home-based training program (150 minutes of walking per week, occurring on three non-consecutive days) using digital tablets. Cognitive behavioral strategies will be implemented to promote physical activity (intervention group). The control group receives a paper-based individualized and progressive home exercise program instruction (2-3 times weekly) without additional support.

***Outcome measures***: Primary: six minute walk test [change at 10 weeks]. Secondary: gait parameters single and dual-task conditions (stride length, steps/min with inertial sensors), Parkinson’s Disease Questionnaire-39 (PDQ-39), physical activity measured with accelerometer (steps per day and time in different intensities), dual task-ability during walking (Auditory Stroop test), balance (MiniBest), executive function (trail making test).

***Comments***: This study will add insights whether an eHealth delivered exercise program will be feasible in promoting physical activity. Although it is not reported to be an outcome, this study may provide insights into possible barriers related to remotely delivering an eHealth intervention to an older PD population with potentially limited digital skills, as having the skill to operate a digital tablet is not an inclusion criterion. Two inclusion criteria seem to contradict each other, namely ’the ability to ambulate indoors without mobility aid’ and ‘the ability to walk continuously for at least 6 minutes with or without a walking aid’. The outcomes are described to be measured as a change in 10 weeks, but the exact time points of theses assessments is not reported. It is unclear whether the trial is powered, or if a convenience sample of 100 participants is determined. The short study period may be insufficient to find evidence on the efficacy of this intervention.

***Results***: The estimated study completion date is in March 2024

***References***: -

**Boston University Charles River Campus - Amplifying Physical Activity Through Music in Parkinson Disease (Amped-PD)**

***Title***: Amped-PD: Amplifying Physical Activity Through a Novel Digital Music Therapeutic in Parkinson Disease

***Phase***: N/A

***Objective***: Study the efficacy of adaptive rhythmic auditory stimulation (RAS) on physical activity levels

***Status***: Recruiting

***Clinicaltrials.gov ID***: NCT05421624

***Sponsor***: Boston University Charles River Campus

***Collaborators***: University of New England

***Estimated enrolment***: 44

***Estimated completion date***: April 2024

***Study design***: This study is a single-blind (outcomes assessor) randomized controlled trial. Participants (*n* = 44) are randomly assigned (parallel assignment 1: 1) to a self-directed walking program with digital music-adaptive rhythmic auditory stimulation (intervention group) or a self-directed walking program (control group). Assessments are performed at baseline, day 4, at the end of the intervention (6 weeks) and up to 2 weeks post-intervention (8 weeks). The trial is recruiting people diagnosed with idiopathic PD, Hoehn and Yahr I-III, age 40 to 80 years, who are community dwelling, have stable PD medication 2 weeks prior to the study, are able to walk independently without aid for at least 10 minutes and can provide authorization to allow communication with the primary healthcare provider for communication. People who have moderate- or significantly disturbing freezing, experienced more than one fall over the past 3 months, have a hearing or cognitive impairment, have cardiac or orthopedic conditions which may limit safe participation, or walk more than 3 times per week for 30 minutes per session, will be excluded.

***Intervention***: Six-week community-based, self-directed walking program with a novel digital device that delivers music-adaptive rhythmic auditory stimulation, comprised of foot sensors, a smartphone app and headphones. The control group receives a 6-week self-directed walking program without rhythmic auditory stimulation.

***Outcome measures***: Primary: physical activity (moderate intense walking; mean number of minutes per day with > 100 steps/min) [baseline, day 4, 6 weeks, 8 weeks], gait quality (variability stride length and swing time) [baseline, 6 weeks, 8 weeks], self-report behavioral automaticity index [baseline, 6 weeks, 8 weeks]. Secondary: 10 meter walk test (10MWT) [baseline, 6 weeks, 8 weeks], 6 minute walk test (6MWT) [baseline, 6 weeks, 8 weeks], walking cadence in-clinic [baseline, 6 weeks, 8 weeks], gait velocity in-clinic [baseline, 6 weeks, 8 weeks], stride length in-clinic [baseline, 6 weeks, 8 weeks], step activity (daily steps) [baseline, day 4, 6 weeks, 8 weeks], Movement Disorders Society Unified Parkinson Disease Rating Scale III (MDS-UPDRS III) [baseline, 6 weeks, 8 weeks], self-efficacy of walking-duration [baseline, 6 weeks, 8 weeks], geriatric depression scale [baseline, 6 weeks, 8 weeks], PDQ-39 [baseline, 6 weeks, 8 weeks], Mini BESTest [baseline, 6 weeks, 8 weeks].

***Comments***: This study is the only study in this overview exploring the effects of auditory stimulation on physical activity. Interestingly, outcomes will be measured 2 weeks post-interventions to assess whether potential effects retain after the intervention. However, the complete study duration is quite short, which may impede evidence based conclusions on the efficacy of this intervention.

***Results***: The estimated study completion date is in April 2024

***References***: -

**University of Pennsylvania - Move to Improve Physical Activity in Parkinson’s Disease (MoTIvatE)**

***Title***: Move to Improve Physical Activity in Parkinson’s Disease: MoTIvatE PD

***Phase***: N/A

***Objective***: Test feasibility and impact of a behaviorally designed gamification intervention to improve goal-directed behavior assessed by physical activity levels

***Status***: Active, not recruiting

***Clinicaltrials.gov ID***: NCT04051151

***Sponsor***: University of Pennsylvania

***Collaborators***: N/A

***Estimated enrolment***: 84

***Estimated completion date***: December 2023

***Study design***: This study is an open-label randomized controlled trial. Participants (*n* = 84) are randomly assigned (parallel assignment 1: 1) to a 4-week gamified exercise intervention together with a partner, including a Fitbit promoting physical activity or standard of care educational resources. Assessments are performed at 4 weeks. The trial was recruiting people diagnosed with PD, Hoehn and Yahr not specified, who are mobile (walking cane allowed) and have a study partner of their choosing. People who have cognitive impairment (Montreal Cognitive Assessment; MoCA < 22), require a wheelchair or walker or are unable to walk safely, are already participating in a physical activity study or are pregnant are excluded.

***Intervention***: Four-week gamification delivered exercise intervention collaborating with a partner, including a Fitbit promoting physical activity, setting daily step goal, point deductions for failure to meet the step goal with progressing or regression of levels based on physical activity. The control group receives standard of care educational sources on importance of physical activity in PD.

***Outcome measures***: Primary: physical activity (steps/day) intervention group compared to control [4 weeks], ability of behavioral phenotyping to predict step goal achievement and change in inactivity (Among the intervention group, compare number of days that step goals were achieved between individuals with greater motivation deficits and those with initiation and planning deficits) [4 weeks].

***Comments***: This study is the only study that involves a partner as a potential motivator in reaching the step goal and enhancing physical activity. This will give important insights in the potential usefulness of this strategy. However, the study period may be rather short. The exact step goal is not reported, which makes it hard to get an idea of the volume of activities participants have to perform. This study plans to relate a deficit in motivation, initiation or planning to their daily step goals, although the way these behavior types will be assessed is not reported.

***Results***: The estimated study completion date is December 1, 2023.

***References***: -

**Radboud University Medical Center - STEPWISE Parkinson: A Smartphone Based Exercise Solution for Patients With Parkinson’s Disease (STEPWISE)**

***Title***: STEPWISE Parkinson: A Smartphone Based, Titrated Exercise Solution for Patients With Parkinson’s Disease in Daily Life

***Phase***: N/A

***Objective***: Study if a smartphone app can increase physical activity over a long period of time, effects of physical activity on fitness, motor- and non-motor symptoms and whether a dose-response relationship exists.

***Status***: Recruiting

***Clinicaltrials.gov ID***: NCT04848077

***Sponsor***: Radboud University Medical Center

***Collaborators***: ZonMw: The Netherlands Organization for Health Research and Development; Massachusetts General Hospital; Hogeschool van Arnhem en Nijmegen (HAN); Canisius-Wilhelmina Hospital; IJsfontein Health BV

***Estimated enrolment***: 452

***Estimated completion date***: July 2026

***Study design***: The study is a double-blind multi-arm, randomized controlled trial. Participants (*n* = 452) are randomly assigned (parallel assignment 1: 1: 1: 1) to one of four groups. All participants will be given access to the STEPWISE motivational application in which participants will be encouraged to increase their physical activities for one year. Different treatment arms will receive a different physical activity goal namely a very high dose, high dose, intermediate dose or low dose (active control group). Assessments are performed at baseline and at the end of the study (week 52). The trial is recruiting people diagnosed with idiopathic PD, Hoehn and Yahr I-III, able to walk independently, understand the Dutch language, who perform equal to or less than 120 minutes of sports/outdoor activity per day (LASA Physical Activity Questionnaire (LAPAQ)), take less than 7000 steps per day during four weeks prior to the study. People who report weekly falls in the previous 3 months, medical conditions that hamper mobility other than PD, live in a nursing home, have cognitive impairments that hamper the use of the motivational app or are not in possession of a suitable smartphone, will be excluded.

***Intervention***: All groups get access to the STEPWISE motivational application in which participants will get feedback and support via the app. They will be encouraged to increase their long-term physical activity, with different proportional increases namely: 1) very high dose 2) high dose 3) intermediate dose. The active control group is assigned to a small proportional increase.

***Outcome measures***: Primary: Physical activity (change in step count per day) [week -4 to 0 and week 49 until 52]. Secondary: Change in physical fitness (6MWT and VO2max) [week 0 and week 53], Parkinson symptoms (MDS-UPDRS) [week 0 and week 53], mobility (TUG) [week 0 and week 53], balance (Mini-BestTest) [week 0 and week 53], gait speed (10MWT) [week 0 and week 53], fear of falling (FES-I) [week 0 and week 53], number of falls (monthly), handgrip strength [week 0 and week 53], self-reported physical activity level (LAPAQ) [week -4, week 0 and week 53], cognition (MoCA) [week 0 and week 53], depression and anxiety (HADS) [week 0 and week 53], apathy (AES-12PD) [week 0 and week 53], fatigue (FSS) [week 0 and week 53], sleep problems and daytime sleepiness (SCOPA-SLEEP) [week 0 and week 53], autonomic dysfunction (SCOPA-AUT) [week 0 and week 53], PDQ-39 [week 0 and week 53], perceived effect of intervention (GPE) [Week 53], System Usability Scale (SUS) [Week 53], perceived physical ability (LIVAS), STEPWISE application data (total time app is used, number of interactions with the app) [week 0 and week 53], adherence [week 0 and week 53], barriers and motivators to engage in physical activity [week 0 and week 53], physical activity measurement with wearable sensor (Axivity AX6) [week 0 and week 53], PD symptoms with the mPower app [Every three months from week 0 to week 53]

***Comments***: This study has the largest sample size in this overview with a long follow-up duration of 12 months. Another interesting aspect is that different volumes of physical activity will be compared allowing for a dose-response analyses. The downside is that the trial does not include a control group that does not receive the motivational application. The study mainly aims for feasibility. Despite the large sample and long follow-up, the efficacy on Parkinson related symptoms will be assessed as secondary outcomes.

***Results***: The estimated study completion date is July 2026.

***References***: -

**University of Cambridge - KEEP Intervention for People Newly Diagnosed With Parkinson’s (KEEP)**

***Title***: KEEP- A Randomised Feasibility Study of a Co-designed Physical Health Education Intervention to Improve Knowledge, Exercise Efficacy and Participation for Newly Diagnosed People With Parkinson’s.

***Phase***: N/A

***Objective***: Study the feasibility of an online delivered, co-designed education intervention

***Status***: Active, not recruiting

***Clinicaltrials.gov ID***: NCT05253040

***Sponsor***: University of Cambridge

***Collaborators***: N/A

***Estimated enrolment***: 30 Estimated completion date: June 2023

***Study design***: The study is a single-blind (outcomes assessor) randomized controlled trial. Participants (*n* = 30) are randomly assigned (parallel assignment 1: 1) to online education modules and participation in online group discussions with a specialized neuro-physiotherapist, or usual care with an education booklet. Assessments are performed at baseline, post-intervention [8 weeks] and at 6 months post-intervention. The trial recruits people diagnosed with PD within 12 months of diagnosis, with stable PD medication for four weeks prior to initiation of the trial and have the ability to understand written English. People who report acute illness, other neurological disease, have dementia or significant cognitive impairments or participate in National Health Service structured PD-specific education program with or without exercise in the last year are excluded.

***Intervention***: Eight-week online education modules and participation in online group discussions with a specialized neuro-physiotherapist. The control group receives usual care with an education booklet on exercise by Parkinson’s UK. Outcome measures: Primary: Recruitment rate (% of eligible participants enrolled) [Through study completion; 12 months], compliance (% of online education sessions completed) [Through study completion; 12 months], drop-out rate [Through study completion; 12 months], acceptability (questionnaire) [Completed at the end of participation in the study; after 8 weeks], feasibility of delivering online intervention (technical problems or difficulties) [Through study completion; 12 months]. Secondary: UPDRS III [Baseline (week 0) and follow-up (6-month post intervention)], MiniBESTest [Baseline (week 0) and follow-up (6-month post intervention)], five time sit to stand [Baseline (week 0) and follow-up (6-month post intervention)], self-efficacy for exercise (SEE) [Baseline (week 0), post-intervention (8 week) and follow-up (6-month post intervention)], HADS [Baseline (week 0), post-intervention (8 week) and follow-up (6-month post intervention)], Apathy evaluation scale (AES) [Baseline (week 0), post-intervention (8 week) and follow-up (6-month post intervention)], multidimensional outcome expectation for exercise scale (MOEES) [Baseline (week 0), post-intervention (8 week) and follow-up (6-month post intervention)], Oxford Participation and Activities Questionnaire [Baseline (week 0), post-intervention (8 week) and follow-up (6-month post intervention)], Recent Physical Activity Questionnaire (RPAQ) [Baseline (week 0), post-intervention (8 week) and follow-up (6-month post intervention)], knowledge of exercise and physical activity questionnaire [Baseline (week 0), post-intervention (8 week) and follow-up (6-month post intervention)], wrist-worn accelerometer (GeneActiv monitor over 7 consecutive days) [Baseline (week 0), post-intervention (8 week) and follow-up (6-month post intervention)]

***Comments***: This study is the only study that provides an intervention solely based on education on the importance of physical activity, rather than providing some sort of exercise program or other cognitive-behavioral strategies like self-monitoring or goal-setting. This study will give insights into how effective only education is in motivating people with PD to engage in more physical activity in daily life. Unfortunately, the number of education modules and online group discussions is not specified. Multiple primary endpoints are reported, which is unusual, and the intervention period of 8 weeks is rather short. However, this study will assess physical activity after a post-intervention period (6 months), which will inform us about retention in the long-term.

***Results***: The estimated study completion date is June 18, 2023

***References***: -

**Boston University Charles River Campus - Walking and mHealth to Increase Participation in Parkinson Disease (WHIP-PD)**

***Title***: Walking and mHealth to Increase Participation in Parkinson Disease

***Phase***: 2

***Objective***: Study the efficacy of an mHealth delivered individualized exercise program with cognitive behavioral elements on physical activity levels

***Status***: Recruiting

***Clinicaltrials.gov ID***: NCT03517371

***Sponsor***: Boston University Charles River Campus

***Collaborators***: Washington University School of Medicine

***Estimated enrolment***: 148 Estimated completion date: May 2024

***Study design***: The study is a single-blind (outcomes assessor), 1-year randomized controlled trial. Participants (*n* = 148) are randomly assigned (parallel assignment 1: 1) to a mHealth delivered exercise program consisting of walking, strengthening and stretching exercises with cognitive-behavioral elements (intervention group) or home-based exercise program that is equally dosed and frequent, but without technological support (control group). Assessments are performed at baseline and the end of the intervention (12 months). The trial is recruiting people diagnosed with idiopathic PD, Hoehn and Yahr I-III, with stable PD medication 2 weeks prior to study entry. People younger than 18 years of age, who are pregnant, have moderate or significantly disturbing freezing (New Freezing of gait questionnaire≥2), cognitive impairment (Mini-Mental State Examination < 24) [19], are unable to walk independently without aid for at least 10 minutes, have cardiac or orthopedic conditions which may limit safe participation, have an unstable medical or psychiatric condition which in the opinion of the investigators would preclude successful participation, live in an institution or medical facility or are engaging in a walking program for more than 90 minutes per week or an exercise regime of moderate intensity for more and 90 minutes per week, will be excluded [19].

***Intervention***: Up to 10 in-person visits with a physical therapist over 12 months supported by a mobile health (mHealth) app delivered home exercise program (walking, strengthening, stretching). At least five exercises on five days per week are prescribed, supported by cognitive-behavioral elements implemented in the mHealth app (goal setting, action planning, automated rewards, self-monitoring of progress) and remote connection to a physical therapist. The control group receives 10 in-person visits with a physical therapist with a home exercise program at the same frequency but without mHealth technology.

***Outcome measures***: Primary: Walking activity (steps/per day) [baseline, 3 months, 6 months and 12 months], walking intensity (number of minutes with > 100 steps) [baseline, 3 months, 6 months and 12 months]. Secondary: walking capacity (6MWT; 10MWT) [baseline, 3 months, 6 months and 12 months], self-efficacy of walking duration [baseline, 3 months, 6 months and 12 months] and barriers self-efficacy scale [baseline, 3 months, 6 months and 12 months]

***Comments***: This study is one of two longest interventions with a follow-up period of 12 months. The WHIP-PD study will show whether motivational reinforcement through a mobile device is of added value on top of a personalized exercise program. They exclude people who walk or exercise for more than 90 minutes per week, which might bias their results. On the other hand, with this criterion they include people who are more likely to benefit from additional motivational support to engage in physical activity. Barriers for engaging in physical activity are monitored on a confidence scale. This intervention comprises an exercise program provided after supervised physical therapy sessions. The motivational intervention is delivered remotely, but is preceded by supervised physical therapy. Complete remote interventions may be even more accessible. Just like STEPWISE, there is no control group receiving no intervention at all, which hinders conclusion solely pointed to the promoting intervention. In addition, this may cause smaller contrast between groups which may lead to a potentially smaller effect.

***Results***: The estimated study completion date is May 31, 2024

***References***: Rawson KS, Cavanaugh JT, Colon-Semenza C, DeAngelis T, Duncan RP, Fulford D, LaValley MP, Mazzoni P, Nordahl T, Quintiliani LM, Saint-Hilaire M, Thomas CA, Earhart GM, Ellis TD. Design of the WHIP-PD study: a phase II, twelve-month, dual-site, randomized controlled trial evaluating the effects of a cognitive-behavioral approach for promoting enhanced walking activity using mobile health technology in people with Parkinson-disease. BMC Neurol. 2020 Apr 20;20(1):146. doi: 10.1186/s12883-020-01718-z.
